# MutS homologue hMSH5: role in cisplatin-induced DNA damage response

**DOI:** 10.1186/1476-4598-11-10

**Published:** 2012-03-08

**Authors:** Joshua D Tompkins, Xiling Wu, Chengtao Her

**Affiliations:** 1 School of Molecular Biosciences, College of Veterinary Medicine, Washington State University, Mail Drop 64-7520, Pullman, WA 99164, USA

**Keywords:** hMSH5, hMSH4, c-Abl, Cisplatin, Homologous recombination

## Abstract

**Background:**

Cisplatin (*cis*-diamminedichloroplatinum (II), CDDP) and its analogues constitute an important class of anticancer drugs in the treatment of various malignancies; however, its effectiveness is frequently affected by mutations in genes involved in the repair and signaling of cisplatin-induced DNA damage. These observations necessitate a need for a better understanding of the molecular events governing cellular sensitivity to cisplatin.

**Results:**

Here, we show that hMSH5 mediates sensitization to cisplatin-induced DNA damage in human cells. Our study indicates that hMSH5 undergoes cisplatin-elicited protein induction and tyrosine phosphorylation. Silencing of hMSH5 by RNAi or expression of hMSH5 phosphorylation-resistant mutant hMSH5^Y742F ^elevates cisplatin-induced G2 arrest and renders cells susceptible to cisplatin toxicity at clinically relevant doses. In addition, our data show that cisplatin promotes hMSH5 chromatin association and hMSH5 deficiency increases cisplatin-triggered γ-H2AX foci. Consistent with a possible role for hMSH5 in recombinational repair of cisplatin-triggered double-strand breaks (DSBs), the formation of cisplatin-induced hMSH5 nuclear foci is hRad51-dependent.

**Conclusion:**

Collectively, our current study has suggested a role for hMSH5 in the processing of cisplatin-induced DSBs, and silencing of hMSH5 may provide a new means to improve the therapeutic efficacy of cisplatin.

## Background

Despite being members of the MMR protein family, the MSH5 homologues have not been demonstrated to function in MMR. Instead, studies in mice, *C. elegans*, and *S. cerevisiae *have shown that MSH5 plays an array of diverse functions ranging from meiotic recombinational DSB repair, maintenance of chromosome integrity, to DNA damage response [1-6]. Purified hMSH4-hMSH5 protein complexes have been shown to possess binding activities towards recombination intermediate structures including the Holliday junction [7], and endogenous hMSH5 has been shown to interact with a Holliday junction binding protein [8]. In addition, hMSH5 forms chromosomal foci in human fetal oocytes at different stages of meiotic prophase I [9].

Coherent with a conjectured role in recombinational DSB repair, hMSH5 has been reported to interact with several proteins related to DSB sensing and repair, including the c-Abl tyrosine kinase and HR protein hRad51 [10,11]. It is observed that RAD51 silencing in MSH5-deficient *C. elegans *oocytes can result in chromosome fragmentation [6], suggesting that MSH5 and RAD51 may play a synergistic role in DSB processing at least during meiosis in *C. elegans*. In addition, interaction between endogenous hMSH5 and hMRE11 has been observed in human alveolar basal epithelial derived lung adenocarcinoma A549 cells [8]. Studies performed with mouse models and human patient samples have also suggested a role for hMSH5 in class switch recombination during B and T cell development, whereas hMSH5 deficiency associates with long microhomologies at Ig switch joints [12]. These observations have raised the possibility that, through interacting with various DSB repair proteins, hMSH5 could exert multiple roles in DNA damage surveillance and DSB repair. Although the link between hMSH5 mutation and diseases in humans has not been explored, a genome-wide association study has designated the *hMSH5 *locus at 6p21.33 as a high risk factor for lung cancer development [13]. In addition to its potential role in DNA repair, hMSH5 interplays with c-Abl in mediating apoptotic response in cells treated with ionizing radiation--a process involved with the activation of p73 and caspase-3 [14].

In the present study, we have investigated the role of hMSH5 in cisplatin-induced DNA damage response. Cisplatin is the drug of choice for combination chemotherapy of testicular cancers [15], and hMSH5 is known to be expressed abundantly in the testis [16]. The cytotoxicity of cisplatin is mainly caused by its ability to form adducts with DNA. The major types of biologically active cisplatin adducts are 1,2-intrastrand crosslinks between guanines or between guanine and adenine, and, to a lesser extent, interstrand DNA crosslinks [17]. These DNA distortions can effectively block the progression of DNA replication and activate cell cycle checkpoint [18-20]. Cisplatin-induced DNA intrastrand crosslinks can be effectively removed by nucleotide excision repair (NER) [17,21]; however, the removal of cisplatin-induced interstrand crosslinks requires the HR pathway [22]. The importance of recombinational repair in resolving cisplatin-induced DNA damage has also been suggested by the observation that cisplatin increases the rate of recombination, presumably attributing to the formation of DSB [23,24]. In addition, the single-strand breaks generated from the processing of cisplatin-induced DNA lesions by NER can also be converted into one-ended DSBs when single-strand breaks collide with the replication forks [25]. Nevertheless, it becomes increasingly clear that HR plays an essential role in the repair of cisplatin-induced DSBs that may arise from stalled replication forks. In addition, HR-deficient cells are highly sensitive to DNA damaging agents that induce DNA cross-links, due to replication blockade.

## Results

### Cisplatin leads to hMSH5 induction

**Figure 1:**
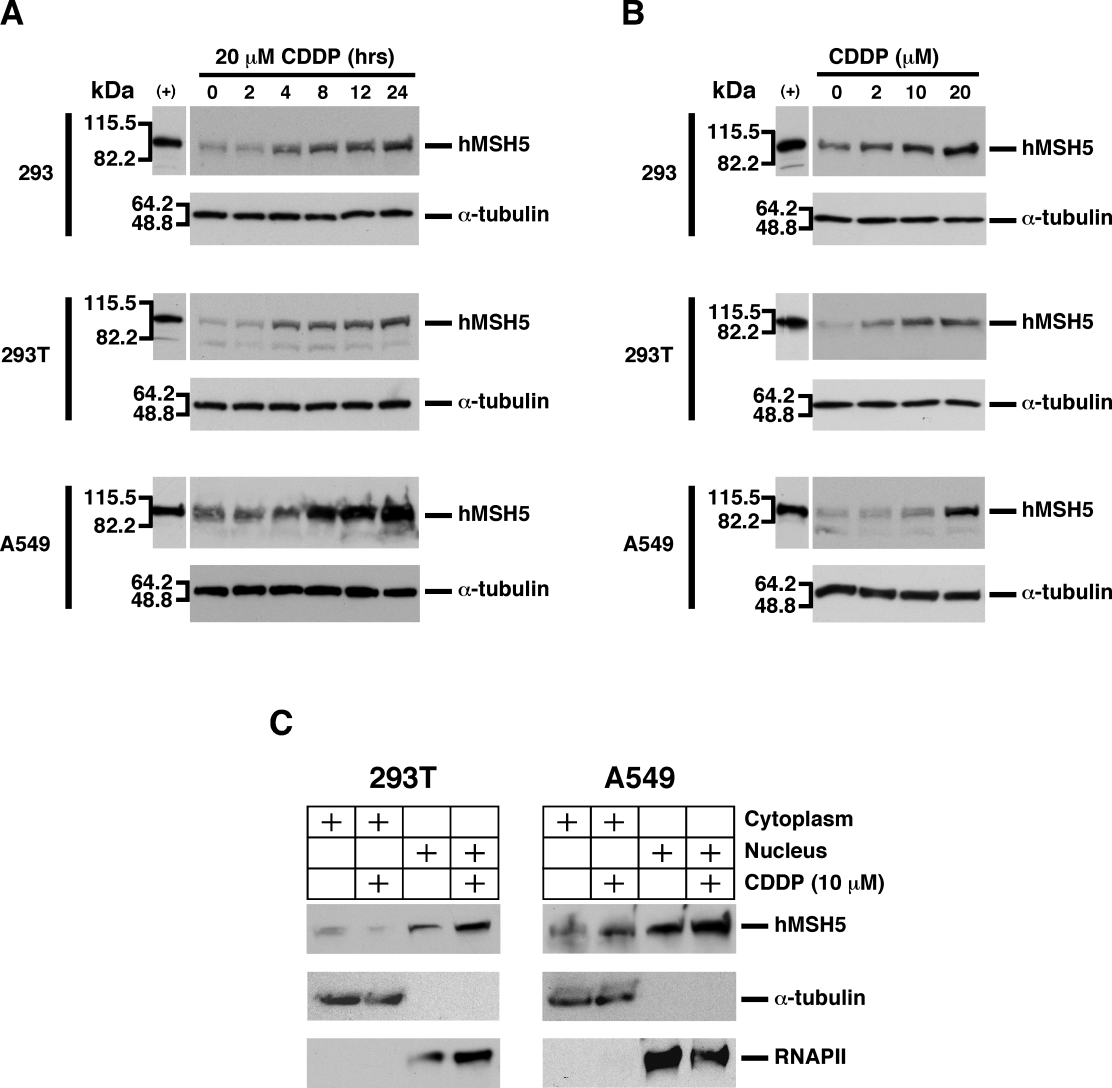
**Cisplatin-mediated hMSH5 protein induction**. (**A**) Time course of hMSH5 protein induction by cisplatin. 293, 293T, and A549 cells were subjected to 20 μM cisplatin and hMSH5 levels were analyzed by Western blotting at the indicated time points. (**B**) Cisplatin dose-dependent induction of hMSH5 protein in 293, 293T and A549 cells. Cells were treated with 2, 10 and 20 μM cisplatin for 24 hrs, and the levels of hMSH5 were analyzed by Western blotting. (+) Lysates from 293T/f-hMSH5 cells were used as positive controls, and α-tubulin was used as a loading control. (**C**) Cisplatin-induced hMSH5 induction occurred predominately in the nucleus. 293T and A549 cells were first treated with 10 μM cisplatin for 8 hrs, followed by Western blot analysis of hMSH5 levels in the nuclear and cytoplasmic fractions. RNAPII and α-tubulin were used as nuclear and cytoplasmic markers, respectively.

Analysis of the effects of cisplatin on the levels of hMSH5 expression in three cell lines--293, 293T, and A549--showed that cisplatin promoted hMSH5 induction in a time- and dose-dependent manner (Figure 1A and 1B). Significant hMSH5 induction could be observed 4 hrs post-treatment by as low as 2 μM cisplatin; in comparison to 293 and 293T cells, hMSH5 induction in A549 cells appeared to be more robust in response to higher dose of cisplatin (Figure 1A and 1B). Since over-expressed hMSH5 can be localized in both cytoplasm and nucleus [26,27], we next examined cisplatin-triggered hMSH5 cytoplasmic and nuclear redistribution in 293T and A549 cells. Immunoblotting analysis demonstrated that the endogenous hMSH5 protein was expressed in both cytoplasm and nucleus, of which the nuclear fraction displayed a moderate level of hMSH5 induction in response to cisplatin treatment in 293T and A549 cells (Figure 1C).

**Figure 2:**
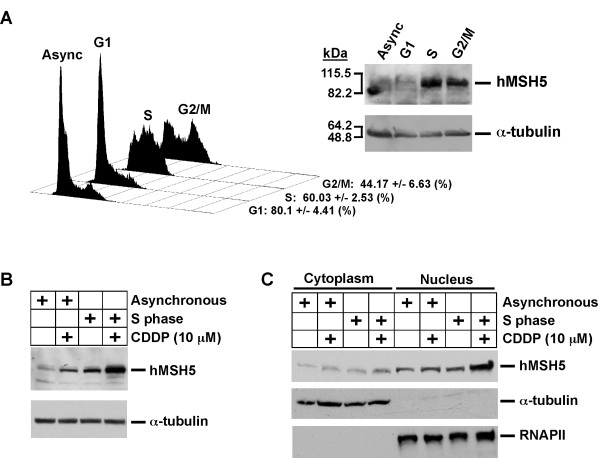
**Cell cycle-dependent hMSH5 expression and cisplatin-triggered hMSH5 protein induction**. (**A**) FACS analysis of cell cycle synchronization. Data were analyzed by the use of FlowJo software (Dean-Jett model). Values are means ± standard deviation from 3 independent measurements. The expression of hMSH5 in 293T cells synchronized to G1, S, G2/M phases, and asynchronized controls were examined by Western blot analysis. α-Tubulin immunoblot was used as a loading control. (**B**) Cisplatin-triggered hMSH5 induction in S phase cells. 293T cells were synchronized at S phase and treated with 10 μM cisplatin for 8 hrs and followed by Western blot analysis of hMSH5 protein levels. α-Tubulin immunoblot was used as a loading control. (**C**) Treatment of S phase cells with cisplatin enhanced hMSH5 nuclear accumulation. Cells synchronized at S phase were treated with 10 μM cisplatin for 8 hrs, and hMSH5 protein levels in the nuclear and cytoplasmic fractions were evaluated by Western blotting. RNAPII and α-tubulin were used as nuclear and cytoplasmic loading controls, respectively.

It is well known that HR is required for the resolution of cisplatin-induced DSBs [22-24], and most of the cisplatin-induced DSBs are created at replication forks during S phase [28]. Interestingly, in synchronized cells, hMSH5 was predominantly expressed in the S and G2 phases of the cell cycle, of which cells in the S phase showed the most abundant hMSH5 expression (Figure 2A). Evidently, following cisplatin treatment, a significant hMSH5 induction was observed in S phase cells (Figure 2B). The results of cytoplasmic/nuclear fractionation experiments were consistent with the view that most of the induction occurs in the nucleus of S phase cells (Figure 2C). Together, these observations raise a possibility that hMSH5 may be involved in the processing of cisplatin-induced DSBs.

### c-Abl phosphorylates hMSH5 at Tyr^742 ^in response to cisplatin-induced DNA lesions

**Figure 3:**
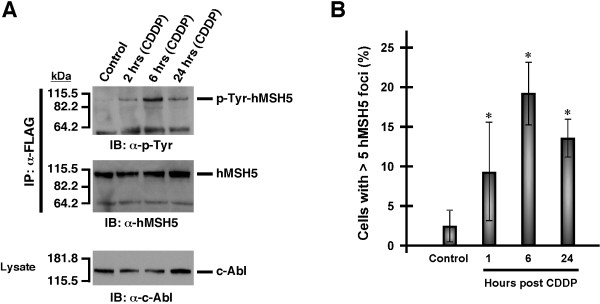
**Cisplatin triggered c-Abl-mediated hMSH5 tyrosine phosphorylation and hMSH5 nuclear foci formation**. (**A**) Cisplatin treatment led to hMSH5 tyrosine phosphorylation. 293T/f-hMSH5 cells were transiently transfected with myc-c-Abl and subjected to 20 μM cisplatin treatment at 48 hrs post-transfection. Cell lysates were then prepared at indicated time points, and the status of tyrosine phosphorylation of immunoaffinity-purified f-hMSH5 was analyzed by Western blotting. Untreated cells were used as controls. (**B**) Time course of cisplatin-induced hMSH5 nuclear foci formation. 293T cells were treated with 10 μM of cisplatin for 2 hrs and hMSH5 foci were examined at 1, 6, and 24 hrs post-treatment. Untreated cells were included as controls. Cells containing five or more hMSH5 nuclear foci were quantified. Error bars represent standard deviations from the means of three independent measurements. Asterisks denote statistical significance (p < 0.05, Student *t*-test) In order to assess the role of hMSH5 tyrosine phosphorylation in this process, we determined the tyrosine residue that can be phosphorylated by c-Abl. To this end, Tyr-to-Phe mutations were introduced to the truncated c-Abl substrate hMSH5 cp-1 [10,29], and each of the resulting hMSH5 cp-1 mutants was co-expressed with c-Abl in BL21(DE3)-RIL cells and subsequently affinity-purified. Immunoblotting analysis of soluble fractions of cell lysates and the affinity-purified hMSH5 cp-1 mutant proteins demonstrated that Tyr-to-Phe mutation at hMSH5 Tyr^742 ^resulted in a complete abolishment of tyrosine phosphorylation, demonstrating that hMSH5 Tyr^742 ^is responsible for c-Abl-mediated phosphorylation (Figure 4A). It is of note that Tyr^742 ^is conserved between human and mouse, and it is located within the hMSH5 domain that interacts with hMSH4 [29,30]. Consistent with this, hMSH5 tyrosine phosphorylation is known to negatively regulate its interaction with hMSH4 [10].

We have previously shown that the interaction between hMSH5 and the c-Abl kinase could lead to hMSH5 tyrosine phosphorylation and c-Abl activation in response to ionizing radiation (IR) [10,14]. To determine whether the hMSH5 and c-Abl interaction is also relevant to cisplatin-triggered DNA damage response, we analyzed c-Abl-mediated hMSH5 tyrosine phosphorylation in 293T/f-hMSH5 cells treated with cisplatin. Western blot analysis indicated that hMSH5 tyrosine phosphorylation became detectable at 2 hrs, peaked at 6 hrs, and began to fade away at 24 hrs post treatment (Figure 3A). This pattern of hMSH5 tyrosine phosphorylation appeared to be temporally correlated with cisplatin-induced hMSH5 nuclear foci formation (Figure 3B and Additional file 1: Figure S1). Approximately 9% of cells possessing hMSH5 nuclear foci were observed 1 hr after cisplatin treatment, and it was further increased to 19.2% at 6 hrs before declining to 13.7% at 24 hrs (Figure 3B). These results implicated a potential role for hMSH5 tyrosine phosphorylation in the processing of cisplatin-induced DNA lesions.

**Figure 4:**
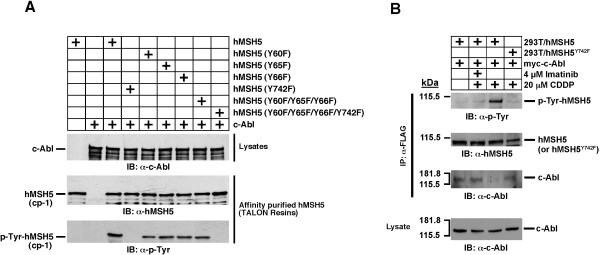
**Identification of hMSH5 tyrosine residues that could be phosphorylated by c-Abl**. (**A**) Six hMSH5 cp-1 mutants, harboring various Tyr-to-Phe mutations, were co-expressed with c-Abl in BL21 cells. The effects of these mutations on tyrosine phosphorylation were examined by immunoblotting analysis of affinity-purified hMSH5 cp-1 proteins with an α-p-Tyr antibody. (**B**) Tyr^742 ^on hMSH5 was targeted by c-Abl kinase in response to cisplatin treatment, and hMSH5 of hMSH5 from c-Abl. 293T/f-hMSH5 and 293T/f-hMSH5^Y742F ^cells were transfected with myc-c-Abl and treated with 20 μM cisplatin 48 hrs post-transfection. Cell lysates were prepared 6 hrs after cisplatin treatment, and α-Flag immunoprecipitates were analyzed by α-p-Tyr and α-c-Abl immunoblots. Four μM imatinib was used to inhibit c-Abl kinase activity.

To validate that Tyr^742 ^could also be targeted by c-Abl in human cells, immunoaffinity-purified hMSH5 proteins from cisplatin-treated and untreated 293T/f-hMSH5 and 293T/f-hMSH5^Y742F ^cells were analyzed for tyrosine phosphorylation. As shown in Figure 4B, cisplatin elicited readily detectable hMSH5 tyrosine phosphorylation, and this phosphorylation was largely absent in cells either expressing hMSH5^Y742F ^or treated with c-Abl kinase inhibitor imatinib. It is known that hMSH5 tyrosine phosphorylation dissociates the binding of hMSH5 to c-Abl [10]. Consistent with this is the observation that hMSH5^Y742F ^could not dissociate from c-Abl following cisplatin treatment (Figure 4B). In addition, hMSH5 Tyr^742 ^phosphorylation could also be triggered by IR (data not shown), suggesting that c-Abl-mediated phosphorylation at hMSH5 Tyr^742 ^is not unique to cisplatin-induced DNA lesions.

### Cisplatin stimulates hMSH5 chromatin association

**Figure 5:**
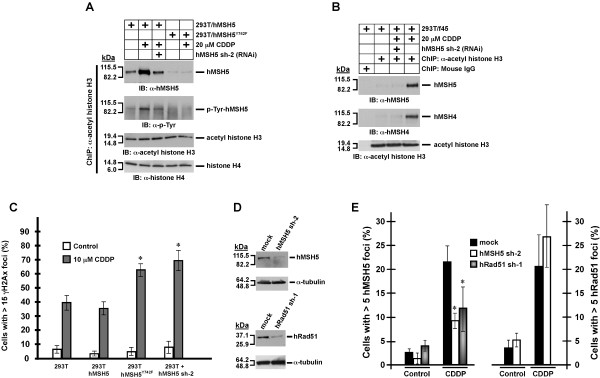
**Analysis of cisplatin-induced hMSH5 association with chromatin and nuclear foci formation**. (**A**) 293T/f-hMSH5 and 293T/f-hMSH5^Y742F ^cells were treated with 20 μM cisplatin, and cross-linked bulk chromatin was immunoprecipitated by an α-acetyl-histone H3 antibody 5 hrs post-treatment. The levels of chromatin-associated hMSH5 and its levels of phosphorylation were analyzed by Western blotting. hMSH5 RNAi was used to knockdown hMSH5. Equal levels of acetyl-histone H3 and histone H4 were present in the immunoprecipitates. (**B**) Analysis of hMSH4 chromatin association following cisplatin treatment. Chromatin was prepared from 293T/f45 cells treated with cisplatin. The levels of chromatin-associated hMSH5 and hMSH4 were analyzed by Western blotting. hMSH5 RNAi was used to knockdown hMSH5. Mouse IgG was used as a negative control. *kDa*, molecular weight (*Mr*) in thousands. (**C**) Examination of γ-H2AX foci formation 24 hrs post cisplatin exposure. 293T, 293T/f-hMSH5, 293T/f-hMSH5^Y742F^, and 293T cells subjected to hMSH5 RNAi were used for this analysis. Cells possessing greater than 15 foci/nucleus were graphically displayed. (**D**) Immunoblotting analysis of the effectiveness of hRad51 or hMSH5 knockdown in 293T cells transfected with pmH1P-Bsd/hRad51 sh-1 or pmH1P-Bsd/hMSH5 sh-2. α-Tubulin was used as a loading control. *kDa*, molecular weight (*Mr*) in thousands. (**E**) Analysis of hMSH5 and hRad51 knockdown on cisplatin-induced hRad51 and hMSH5 nuclear foci formation. Cells were subjected to 10 μM cisplatin for 2 hrs and were analyzed for hMSH5 foci formation 6 hrs post cisplatin removal. Cells that possessed five or more nuclear foci for hMSH5 or hRad51 were scored. Error bars represent standard deviations of the means of three independent measurements. Statistically significant differences between knockdown and control cells were indicated with asterisks (p < 0.05, Student *t*-test).

To analyze cisplatin-triggered hMSH5 chromatin association, ChIP analysis was performed with an α-acetyl histone H3 antibody using 293T/f-hMSH5 and 293T/f-hMSH5^Y742F ^cells. Western blot was employed to determine the levels of chromatin-associated hMSH5 under various conditions. Results of these experiments indicated that cisplatin treatment significantly enhanced the association of hMSH5, but not hMSH5^Y742F^, with chromatin (Figure 5A). Clearly, a fraction of chromatin-bound hMSH5 was tyrosine phosphorylated, and RNAi-mediated hMSH5 silencing lessened the tyrosine phosphorylation signal along with a corresponding reduction on the level of chromatin-associated hMSH5 (Figure 5A), suggesting hMSH5 tyrosine phosphorylation is required for cisplatin-induced hMSH5 chromatin association. Since the hMSH5-hMSH4 heterocomplex has been suggested to play a role in the process of HR [7], we also examined whether cisplatin could trigger hMSH4 chromatin localization. The results of a similar ChIP analysis performed with 293T/f45 cells [31]--expressing both hMSH5 and hMSH4--indicated that cisplatin could trigger hMSH4 localization to chromatin, and this hMSH4-chromatin association was entirely hMSH5-dependent as such RNAi-mediated hMSH5 silencing could diminish hMSH4-chromatin association (Figure 5B). These observations suggest that c-Abl-mediated phosphorylation at hMSH5 Tyr^742 ^is important for cisplatin-induced chromatin association of hMSH5 and hMSH4. Because it is known that tyrosine phosphorylation dissociates hMSH5 from hMSH4 as well as c-Abl [10,14], it is conceivable that chromatin-bound hMSH5 will have to be in the dephosphorylated form before it can interact with hMSH4. This view is consistent with the observed low levels of tyrosine phosphorylation of chromatin-bound hMSH5 (Figure 5A).

### hMSH5 facilitates the repair of cisplatin-induced DNA lesions

The appearance of γ-H2AX foci is an intrinsic biomarker for the extent of DSB formation and can be used to assess the combined effects of DNA damage and DNA lesion repair [32]. It is known that the processing of cisplatin-induced DNA lesions coincides with DSB formation [24]. Therefore, in order to analyze the effects of hMSH5 on the processing of cisplatin-induced DSBs, γ-H2AX foci positive cells were quantified at 24 hrs post-cisplatin treatment. Approximately 39.6% of 293T cells and 35.5% of 293T/f-hMSH5 cells were γ-H2AX foci positive (Figure 5C and Additional file 1: Figure S2). The small difference between these two cell lines indicates that the effects of exogenously over-expressed hMSH5 can be sufficiently masked by cisplatin-triggered induction of endogenous hMSH5 (Figure 5C). However, under identical experimental conditions, expression of hMSH5^Y742F ^or silencing of hMSH5 significantly increased the γ-H2AX positive population to about 62.5% and 69.4%, respectively (Figure 5C), suggesting that hMSH5 deficiency can significantly delay the resolution of cisplatin-induced DSBs.

Since the key HR protein hRad51 coexists with hMSH5 and c-Abl in the same protein complex [10,11], and Rad51 has been implicated in the repair of cisplatin-induced DSBs [33], we next investigated the possibility that hMSH5 functions together with hRad51 in the processing of cisplatin-induced DNA lesions. The results of immunostaining experiments demonstrated that, in response to cisplatin, both hMSH5 and hRad51 formed nuclear foci in both 293T and A549 cells (Additional file 1: Figure S3). To determine whether the formation of cisplatin-triggered hMSH5 foci was hRad51-dependent, RNAi-mediated hMSH5 and hRad51 knockdown were performed (Figure 5D). As shown in Figure 5E, the formation of cisplatin-induced hMSH5 foci was significantly diminished with the knockdown of either hMSH5 or hRad51 (left panel). However, the formation of cisplatin-induced hRad51 foci was not affected by the silencing of hMSH5 (Figure 5E, right panel), suggesting that hMSH5 acts downstream of hRad51 in recombinational processing of cisplatin-induced DSBs.

### hMSH5 deficiency renders cells more sensitive to cisplatin toxicity

**Figure 6:**
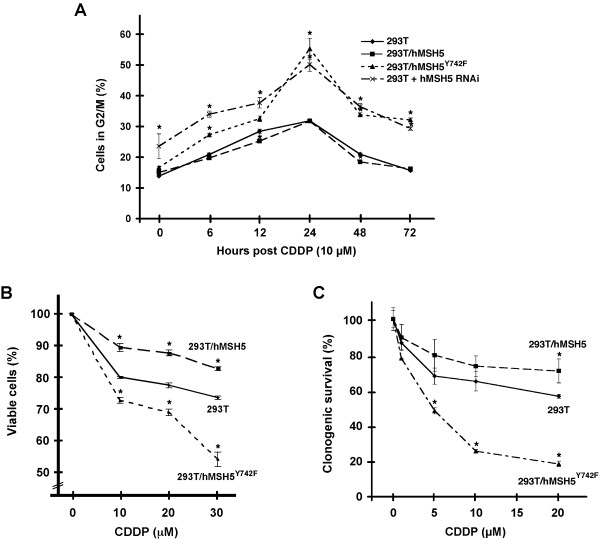
**The effects of hMSH5 deficiency on cisplatin sensitivity**. (**A**) hMSH5 deficiency increased G2/M arrest in response to cisplatin treatment. 293T, 293T/f-hMSH5, 293T/f-hMSH5^Y742F^, and 293T hMSH5 RNAi cells (hMSH5-silenced 293T cells) were treated with cisplatin for 2 hrs, and were used for the cell cycle analysis at indicated time points. Percentages of G2/M cells were determined using FlowJo (Dean-Jett model). (**B**) MTT assays were performed to determine the proliferation of 293T, 293T/f-hMSH5 and 293T/hMSH5^Y742F ^cells in response to cisplatin treatment. (**C**) Clonogenic survival analysis of 293T, 293T/f-hMSH5, and 293T/f-hMSH5^Y742F ^cells treated with different doses of cisplatin. Cells were treated with indicated doses for 1 hr and maintained in culture for 14 days to allow colony formation. Error bars represent standard deviations from the means of three independent measurements. Asterisks denote p < 0.05 by Student *t*-test.

Cisplatin is known to induce G2/M arrest, and defective recombination often renders cells more sensitive to its toxic effects. Therefore, we next analyzed the effects of hMSH5 deficiency on cisplatin-triggered G2/M arrest and survivability. Specifically, cell cycle analysis was performed with cisplatin-treated 293T, 293T/f-hMSH5, 293T/f-hMSH5^Y742F^, and hMSH5-silenced 293T cells over a period of 72 hrs, in which the highest level of G2/M arrest occurred 24 hrs after cisplatin treatment (Figure 6A). The baseline levels of G2/M cells (i.e. at zero hr time point) for 293T, 293T/f-hMSH5, and 293T/f-hMSH5^Y742F ^were identical (Figure 6A). However, there was a slight increase of the basal G2/M population for hMSH5-silenced 293T cells, presumably due to the effects of transient transfection (Figure 6A). Although all cell lines displayed G2/M arrest following cisplatin treatment, cells with hMSH5^Y742F ^or hMSH5 RNAi had significantly larger G2/M populations especially at late time points (Figure 6A). By 24 hrs, 293T/f-hMSH5^Y742F ^and hMSH5 knockdown cells, as compared to 293T cells, increased G2/M arrest by 22.7% and 17.8%, respectively (Figure 6A). Moreover, cells with defective hMSH5 were unable to resume normal cell cycle distribution at 72 hrs post-treatment, while 293T and 293T/f-hMSH5 cells were able to return to normal cell cycle distribution starting at 48 hrs (Figure 6A).

To validate whether cells harboring defective hMSH5 could confer a sensitive phenotype to cisplatin toxicity, MTT assays and clonogenic survival analyses were performed with 293T, 293T/f-hMSH5, and 293T/f-hMSH5^Y742F ^cells treated with cisplatin. Evidently, 293T/f-hMSH5^Y742F ^cells were very sensitive to the toxic effects of cisplatin in both the MTT and clonogenic survival assays, whereas over-expression of hMSH5 appeared to provide a moderate protection to cisplatin toxicity (Figure 6B and 6C). Consistent with the idea that hMSH5 functions together with hRad51 in the process of HR, Rad51-deficient cells have also been shown to exhibit a cisplatin-sensitive phenotype [33].

The dominant negative effects exerted by the phosphorylation-resistant mutant hMSH5^Y742F ^may be attributed to competition between hMSH5^Y742F ^and the endogenous hMSH5 for binding to partner proteins such as c-Abl, hRad51, hMRE11 and hMSH4. This view is supported by the observed normal interaction of hMSH5^Y742F ^with c-Abl or hMSH4 (Additional file 1: Figure S4). Because c-Abl-mediated hMSH5 tyrosine phosphorylation is known to reduce hMSH5 interaction with c-Abl and hMSH4 [10], expression of hMSH5^Y742F ^could also disrupt the dynamic composition of its associated protein complex. Together, this study suggests a role for hMSH5 in recombinational repair of cisplatin-induced DSBs.

## Discussion

Our current study has demonstrated an important role for hMSH5 in the processing of cisplatin-elicited S phase-dependent DSBs. Like other HR repair proteins, hMSH5 is predominantly expressed during S and G2/M phases of the cell cycle. Given the observation that silencing of hRad51 can significantly compromise the formation of cisplatin-induced hMSH5 foci, it is conceivable that hMSH5 acts downstream of hRad51 in the DSB repair process. Disruption of hMSH5 function either by RNAi or by the over-expression of a phosphorylation-deficient mutant significantly increased the number of cells displaying γ-H2AX foci at 24 hrs after cisplatin treatment, indicating an increase in the retention of cisplatin-induced DSBs. In addition, disruption of hMSH5 enhances a sustained cisplatin-triggered G2 arrest, thereby rendering cells more sensitive to cisplatin toxicity. It appears that the reduction of clonogenic survivability correlates well with the levels of γ-H2AX foci in 293T, 293T/f-hMSH5, and 293T/f-hMSH5^Y742F ^cells at 24 hrs after cisplatin challenge. This observation is consistent with previous reports showing that the level of γ-H2AX foci retention 24 hrs after treatment is a useful indicator for cisplatin-mediated cell killing [28,34]. Although the precise role of hMSH5 in this process remains to be delineated, the current evidence collectively supports a scenario by which hMSH5 functions downstream of hRad51 in recombinational repair of cisplatin-induced DSBs.

Due to its radio-sensitizing activity, cisplatin has been frequently used in combination chemoradiation therapy of human malignancies [35]. Presently, however, the mechanisms underlying the effects of this radiosensitizer are still being studied. It is suggested that cisplatin adducts can block the repair of ionizing radiation-induced DSBs by the nonhomologous end-joining pathway [35,36]. However, the results of our current study have raised another possibility that cisplatin-triggered hMSH5 induction may potentially contribute to the effectiveness of cisplatin combination chemoradiation therapy. It is known that higher levels of hMSH5 promote IR-induced apoptosis [14]. Thus, in spite of the fact that cisplatin-triggered hMSH5 induction can facilitate the repair of cisplatin-induced DSBs, the higher levels of hMSH5 could promote a robust apoptotic response to IR during cisplatin combination chemoradiation therapy.

In addition to hMSH5, other MMR proteins are also involved in cisplatin-triggered DNA damage repair and response. In fact, the roles of several other MMR family members in mediating cellular responses to cisplatin-induced DNA lesions have been studied [37-46]. As a whole, these studies highlight two opposite effects of individual MMR proteins on cellular responses to cisplatin toxicity. By functioning in the recognition and signaling of cisplatin-induced DNA lesions, MMR proteins can promote cell killing. On the other hand, they can directly participate in the processing of cisplatin-induced DSBs, thereby exerting a protective effect. It has been shown that hMSH2 recognizes cisplatin-induced DNA lesions through direct binding of DNA-cisplatin adducts [41,42]. Cells defective in the expression of hMSH2 or hMLH1 often exhibit a 2- to 4-fold increase in resistance to cisplatin in comparison to corresponding controls [37,39,40,44-46], indicating that hMSH2 and hMLH1 are involved in mediating cisplatin-triggered DNA damage signaling. Accordingly, cisplatin-resistant cells derived from repetitive drug selection are frequently associated with defective hMSH2 or hMLH1 expression [47-49]. However, the effects of hMSH2 or hMLH1 in cellular sensitization to cisplatin have not been observed in a few other studies, reflecting the complex nature of cellular response to cisplatin-induced DNA damage (reviewed in ref. [19]). It is conceivable that the roles of hMSH2 and hMLH1 may be regulated differently in the processes of repair and DNA damage signaling in different cell types. In addition, difference in cell cycle regulation may be another important factor in controlling various levels of cellular sensitivity to cisplatin. In fact, it is demonstrated recently that disruption of RPA's role in cell cycle regulation synergistically enhances the cytotoxic effects of cisplatin [50].

In contrast to aforementioned observations, a recent study has revealed an important role for hMSH3 in the repair of cisplatin-induced DSBs [38]. Using an isogenic HCT116-derived cell line in which the expression of hMSH3 can be controlled, Goel and colleagues [38] demonstrated that hMSH3 deficiency sensitizes cells to both cisplatin and oxaliplatin toxicity, and this effect of hMSH3 is not dependent on the canonical MMR pathway. In addition, in response to oxaliplatin treatment, hMSH3-deficient cells sustain a higher level of γ-H2AX, suggesting that hMSH3 plays an important role in DSB repair [38]. Intriguingly, the role of hMSH5 in mediating cellular response to cisplatin-induced DSBs bears a resemblance to that reported for hMSH3. In spite of using different cell lines and different ways to disrupt gene expression, cells deficient in hMSH5 or hMSH3 show comparable levels of reduction in clonogenic survivability in response to the same doses of cisplatin (Figure 6C) [38]. Although the timing for the elevation of treatment-induced γ-H2AX appears to be different in cells subjected to RNAi-mediated silencing of hMSH5 or hMSH3 (Figure 5C) [38], these observations warrant future studies to determine whether these two MutS homologues act in the same repair process of cisplatin-induced DSBs.

Intuitively, fertility preservation in male cancer patients undergoing chemotherapy is highly desired, and the relatively high levels of hMSH5 expression in the testis would be expected to provide a protection against cispaltin-induced DSBs. However, this effect of hMSH5 undoubtedly requires coordinated actions from a network of proteins involved in the repair process, and the efficiency of this pathway in various cell types in the testis is presently unknown. Since cisplatin represents a main treatment choice for testicular cancers [15], it would be interesting to investigate the relative expression levels of hMSH5 in testicular tumors and matched normal testicular tissues. This information will be useful for assessing the value of using hMSH5 as a prognosis biomarker. Finally, our study has implicated that combining hMSH5 disruption with cisplatin treatment might be an alternative strategy for enhancing the therapeutic effects of cisplatin.

## Conclusion

In summary, our study has demonstrated a role for hMSH5 in protecting cells from cisplatin-induced DNA damage. Inactivation of hMSH5 by RNAi or by expressing a phosphorylation-deficient hMSH5 mutant elevates cisplatin-induced G2 arrest and renders cells susceptible to cisplatin toxicity.

Collectively, our data is compatible with the idea that hMSH5 is involved in HR repair of cisplatin-induced DSBs.

## Methods

### Cell lines and cell cultures

Stable cell line 293T/f-hMSH5^Y742F ^was generated by a similar procedure that was previously described for 293T/f-hMSH5 [10]. All human cell lines were maintained in DMEM (Invitrogen, Carlsbad, CA) containing 10% FBS (Biomeda, Foster City, CA) and 1x Penicillin-Streptomycin (Invitrogen). Cisplatin (Cis-diamminedichloroplatinum (II) or CDDP) (Sigma, St. Louis, MO) was used for the induction of DNA damage, and imatinib (Novartis, Basel, Switzerland) was used to inhibit c-Abl kinase activity. Cell synchronization at different cell cycle phases was performed by the use of standard hydroxyurea (Sigma), double thymidine (Sigma), and nocodazole (Sigma) based procedures [37,51]. Cell cycle analysis was performed with the standard propidium iodide (Invitrogen) staining procedure as described previously [52], in which at least 10,000 cells were analyzed by the use of FlowJo V8 (Tree Star Inc., Ashland, OR). Clonogenic survival and MTT analysis were performed by the same procedures described previously [14,52].

### Antibodies, Western blot analysis, and immunoprecipitation (IP)

Antibodies used for performing IP and Western blot analysis included α-FLAG M2 (Sigma, St. Louis, MO), α-hMSH5 [29], α-hMSH4 [53], α-γ-H2AX (Upstate Laboratories, Inc., East Syracuse, NY), α-hRad51 (Calbiochem, Gibbstown, NJ), α-hMRE11 (Novus Biologicals Inc., Littleton, CO), α-c-Abl (BD Pharmingen, San Diego, CA), α-p-Tyr (Cell Signaling, Beverley, MA), α-α-tubulin (Sigma, St. Louis, MO), α-acetyl histone H3 (Upstate), α-histone H4 (Cell Signaling), and α-RNAPII (Upstate). IP and Western blotting were performed as described previously [29].

### Immunofluorescence analysis of nuclear foci

Analysis of hMSH5, hRad51, and γ-H2AX foci formation was carried out by following a standard immunofluorescence protocol. Primary and secondary antibodies used for these experiments included α-hMSH5 [29], α-hRad51 (Ab-1) pAb (Calbiochem), α-hRad51 (14B4) mAb (Novus Biologicals Inc.), α-γ-H2AX (Upstate), Oregon Green goat α-mouse IgG and Texas Red goat α-rabbit IgG (Invitrogen, Carlsbad, CA). Cells were mounted with Vectashield mounting media containing DAPI (Vector Laboratories, Birminham, CA) and visualized by a Zeiss Axioplan fluorescence microscope. For nuclear foci quantification, cell counting was performed at least three times with a total of > 200 cells counted.

### Bacterial Protein expression and purification

Expression of various hMSH5 cp-1 Tyr-to-Phe mutants and c-Abl in BL21(DE3)-RIL cells was performed using the same experimental procedure as described previously [10]. PCR-based site-directed mutagenesis was adapted to generate DNA sequences encoding hMSH5 Tyr-to-Phe mutations. Recombinant hMSH5 cp-1 or hMSH5 cp-1 Tyr-to-Phe proteins were purified under native conditions by the use of TALON Metal Affinity Resins (Clontech, Mountainview, CA) as described previously [10].

### Nuclear/cytoplasmic fractionation

Nuclear and cytoplasmic fractions were prepared by the use of NE-PER Nuclear and Cytoplasmic Extraction Reagents (Pierce, Thermo Fisher Scientific Inc., Rockford, IL) in accordance with the manufacturer's recommendation. Western blots with α-RNAPII (Upstate) and α-α-tubulin (Sigma) antibodies were performed to validate successful fractionation.

### Chromatin association assays

Chromatin immunoprecipitation (ChIP) was performed according to the manufacturer's recommendation (EZ-ChIP kit, Millipore, Billerica, MA). Antibodies used in the experiments included α-acetyl histone H3 (Upstate), mouse IgG (Upstate), α-p-Tyr (Cell Signaling), α-hMSH5 [29], α-hMSH4 [53], and α-histone H4 (Cell Signaling). To analyze chromatin-associated proteins, bulk chromatin was immunoprecipitated with 10 μg of α-acetyl-histone H3. Immunoprecipitates were then subjected to Western blotting analysis.

### RNA interference

The generation of pmH1P-based RNAi constructs was performed as previously described [54]. The efficiency of RNAi-mediated gene silencing was validated by Western blotting, and those displayed greater than 50% knockdown were selected. The RNAi targets were hMSH5 sh-2 [14] and hRad51 sh-1 (5'-AAGGAGAGTGCGGCGCTTC).

## Abbreviations

CDDP: Cisplatin (*cis*-diamminedichloroplatinum (II)); DSB: Double-strand break; MMR: Mismatch repair; HR: Homologous recombination; MSH5: MutS homologue 5; NER: Nucleotide excision repair

## Competing interests

The authors declare that they have no competing interests.

## Authors' contributions

JT carried out most of the experiments and participated in the preparation of the manuscript. XW participated in reagent preparation, interpretation of critical data, and manuscript preparation and revision. CH conceived of the study and participated in its design and coordination as well as manuscript preparation. All authors read and approved the final manuscript.

## Authors' information

School of Molecular Biosciences, Mail Drop 64-7520, College of Veterinary Medicine, Washington State University, Pullman, WA 99164, USA

## Supplementary Material

Additional file 1 **Figure S1 **Representative images of CDDP-induced hMSH5 foci formation at 1, 6, and 24 hrs post treatment. Cells were treated with 10 μM CDDP for 2 hrs and hMSH5 foci formation was analyzed at indicated time points after treatment. **Figure S2**. Representative images of cisplatin-induced γ-H2AX foci formation in 293T, 293T/f-hMSH5, 293T/f-hMSH5^Y742F^, and 293T hMSH5 RNAi cells (48 hrs post transfection with pmH1P-Bsd/hMSH5 sh-2). (A) Untreated cells were examined in parallel to establish the basal levels of γ-H2AX signal in these cell lines. (B) Cells were treated with 10 μM cisplatin for 2 hrs followed by γ-H2AX foci analysis at 24 hrs after cisplatin removal. Nuclei are counterstained with DAPI and merged images are provided. **Figure S3**. Representative images of CDDP-induced γ-H2AX, hRad51, and hMSH5 foci formation in 293T and A549 cells. (A) Analysis of CDDP-triggered γ-H2AX and hMSH5 foci formation. (B) Analysis of CDDP-triggered γ-H2AX and hRad51 foci formation. (C) Analysis of CDDP-triggered hRad51 and hMSH5 foci formation. Consistent with hMSH5 cytoplasmic and nuclear distribution patterns, CDDP-induced hMSH5 foci appear to be present in both cytoplasm and nucleus, whereas CDDP-induced γ-H2AX and hRad51 foci are predominately nuclear. Arrows indicate potential overlaps of two different signals. **Figure S4**. Effects of hMSH5 Tyr^742^-to-Phe mutation on its interaction with hMSH4 and c-Abl. (A) Co-IP analysis of the interaction between hMSH5 cp-1 Y742F and hMSH4. The results indicated that hMSH5 cp-1 Y742F could interact with hMSH4 as efficient as hMSH5 cp-1. (B) Co-IP analysis of the interaction between hMSH5^Y742F ^and c-Abl. hMSH5^Y742F ^interacted with c-Abl in the same manner as hMSH5 did. Click here for file

## References

[B1] de Vries SS Baart EB Dekker M Siezen A de Rooij DG de Boer P te Riele H Mouse MutS-like protein Msh5 is required for proper chromosome synapsis in male and female meiosis Genes Dev 1999 13 523 531 10.1101/gad.13.5.523 10072381 PMC316502

[B2] Edelmann W Cohen PE Kneitz B Winand N Lia M Heyer J Kolodner R Pollard JW Kucherlapati R Mammalian MutS homologue 5 is required for chromosome pairing in meiosis Nat Genet 1999 21 123 127 10.1038/5075 9916805

[B3] Hollingsworth NM Ponte L Halsey C MSH5, a novel MutS homolog, facilitates meiotic reciprocal recombination between homologs in Saccharomyces cerevisiae but not mismatch repair Genes Dev 1995 9 1728 1739 10.1101/gad.9.14.1728 7622037

[B4] Bawa S Xiao W A mutation in the MSH5 gene results in alkylation tolerance Cancer Res 1997 57 2715 2720 9205082

[B5] Bawa S Xiao W A single amino acid substitution in MSH5 results in DNA alkylation tolerance Gene 2003 315 177 182 14557077 10.1016/s0378-1119(03)00737-6

[B6] Rinaldo C Bazzicalupo P Ederle S Hilliard M La Volpe A Roles for Caenorhabditis elegans rad-51 in meiosis and in resistance to ionizing radiation during development Genetics 2002 160 471 479 11861554 10.1093/genetics/160.2.471 PMC1461995

[B7] Snowden T Acharya S Butz C Berardini M Fishel R hMSH4-hMSH5 recognizes Holliday Junctions and forms a meiosis-specific sliding clamp that embraces homologous chromosomes Mol Cell 2004 15 437 451 10.1016/j.molcel.2004.06.040 15304223

[B8] Kato T Sato N Hayama S Yamabuki T Ito T Miyamoto M Kondo S Nakamura Y Daigo Y Activation of Holliday junction recognizing protein involved in the chromosomal stability and immortality of cancer cells Cancer Res 2007 67 8544 8553 10.1158/0008-5472.CAN-07-1307 17823411

[B9] Lenzi ML Smith J Snowden T Kim M Fishel R Poulos BK Cohen PE Extreme heterogeneity in the molecular events leading to the establishment of chiasmata during meiosis i in human oocytes Am J Hum Genet 2005 76 112 127 10.1086/427268 15558497 PMC1196414

[B10] Yi W Lee TH Tompkins JD Zhu F Wu X Her C Physical and functional interaction between hMSH5 and c-Abl Cancer Res 2006 66 151 158 10.1158/0008-5472.CAN-05-3019 16397227

[B11] Her C Zhao N Wu X Tompkins JD MutS homologues hMSH4 and hMSH5: diverse functional implications in humans Front Biosci 2007 12 905 911 10.2741/2112 17127347

[B12] Sekine H Ferreira RC Pan-Hammarstrom Q Graham RR Ziemba B de Vries SS Liu J Hippen K Koeuth T Ortmann W Role for Msh5 in the regulation of Ig class switch recombination Proc Natl Acad Sci USA 2007 104 7193 7198 10.1073/pnas.0700815104 17409188 PMC1855370

[B13] Wang Y Broderick P Webb E Wu X Vijayakrishnan J Matakidou A Qureshi M Dong Q Gu X Chen WV Common 5p15.33 and 6p21.33 variants influence lung cancer risk Nat Genet 2008 40 1407 1409 10.1038/ng.273 18978787 PMC2695928

[B14] Tompkins JD Wu X Chu YL Her C Evidence for a direct involvement of hMSH5 in promoting ionizing radiation induced apoptosis Exp Cell Res 2009 315 2420 2432 10.1016/j.yexcr.2009.05.004 19442657 PMC3171649

[B15] Einhorn LH Curing metastatic testicular cancer Proc Natl Acad Sci USA 2002 99 4592 4595 10.1073/pnas.072067999 11904381 PMC123692

[B16] Her C Doggett NA Cloning, structural characterization, and chromosomal localization of the human orthologue of Saccharomyces cerevisiae MSH5 gene Genomics 1998 52 50 61 10.1006/geno.1998.5374 9740671

[B17] Kartalou M Essigmann JM Mechanisms of resistance to cisplatin Mutat Res 2001 478 23 43 10.1016/S0027-5107(01)00141-5 11406167

[B18] Comess KM Burstyn JN Essigmann JM Lippard SJ Replication inhibition and translesion synthesis on templates containing site-specifically placed cis-diamminedichloroplatinum(II) DNA adducts Biochemistry 1992 31 3975 3990 10.1021/bi00131a013 1314653

[B19] O'Brien V Brown R Signalling cell cycle arrest and cell death through the MMR System Carcinogenesis 2006 27 682 692 16332722 10.1093/carcin/bgi298

[B20] Ciccarelli RB Solomon MJ Varshavsky A Lippard SJ In vivo effects of cis- and trans-diamminedichloroplatinum(II) on SV40 chromosomes: differential repair, DNA-protein cross-linking, and inhibition of replication Biochemistry 1985 24 7533 7540 10.1021/bi00347a005 3004558

[B21] Moggs JG Szymkowski DE Yamada M Karran P Wood RD Differential human nucleotide excision repair of paired and mispaired cisplatin-DNA adducts Nucleic Acids Res 1997 25 480 491 10.1093/nar/25.3.480 9016585 PMC146461

[B22] McHugh PJ Spanswick VJ Hartley JA Repair of DNA interstrand crosslinks: molecular mechanisms and clinical relevance Lancet Oncol 2001 2 483 490 10.1016/S1470-2045(01)00454-5 11905724

[B23] Nowosielska A Calmann MA Zdraveski Z Essigmann JM Marinus MG Spontaneous and cisplatin-induced recombination in Escherichia coli DNA Repair (Amst) 2004 3 719 728 10.1016/j.dnarep.2004.02.009 15177181

[B24] Nowosielska A Marinus MG Cisplatin induces DNA double-strand break formation in Escherichia coli dam mutants DNA Repair (Amst) 2005 4 773 781 10.1016/j.dnarep.2005.03.006 15925551

[B25] Helleday T Lo J van Gent DC Engelward BP DNA double-strand break repair: from mechanistic understanding to cancer treatment DNA Repair (Amst) 2007 6 923 935 10.1016/j.dnarep.2007.02.006 17363343

[B26] Neyton S Lespinasse F Lahaye F Staccini P Paquis-Flucklinger V Santucci-Darmanin S CRM1-dependent nuclear export and dimerization with hMSH5 contribute to the regulation of hMSH4 subcellular localization Exp Cell Res 2007 313 3680 3693 10.1016/j.yexcr.2007.08.010 17869244

[B27] Lahaye F Lespinasse F Staccini P Palin L Paquis-Flucklinger V Santucci-Darmanin S hMSH5 is a nucleocytoplasmic shuttling protein whose stability depends on its subcellular localization Nucleic Acids Res 2010 38 3655 3671 10.1093/nar/gkq098 20185565 PMC2887964

[B28] Olive PL Banath JP Kinetics of H2AX phosphorylation after exposure to cisplatin Cytometry B Clin Cytom 2009 76 79 90 18727058 10.1002/cyto.b.20450

[B29] Yi W Wu X Lee TH Doggett NA Her C Two variants of MutS homolog hMSH5: prevalence in humans and effects on protein interaction Biochem Biophys Res Commun 2005 332 524 532 10.1016/j.bbrc.2005.04.154 15907804

[B30] Her C Wu X Wan W Doggett NA Identification and characterization of the mouse MutS homolog 5: Msh5 Mamm Genome 1999 10 1054 1061 10.1007/s003359901161 10556423

[B31] Lee TH Yi W Griswold MD Zhu F Her C Formation of hMSH4-hMSH5 heterocomplex is a prerequisite for subsequent GPS2 recruitment DNA Repair (Amst) 2006 5 32 42 10.1016/j.dnarep.2005.07.004 16122992

[B32] Olive PL Retention of gammaH2AX foci as an indication of lethal DNA damage Radiother Oncol 2011 10.1016/j.radonc.2011.05.055 21704409

[B33] Takata M Sasaki MS Tachiiri S Fukushima T Sonoda E Schild D Thompson LH Takeda S Chromosome instability and defective recombinational repair in knockout mutants of the five Rad51 paralogs Mol Cell Biol 2001 21 2858 2866 10.1128/MCB.21.8.2858-2866.2001 11283264 PMC86915

[B34] Banath JP Klokov D MacPhail SH Banuelos CA Olive PL Residual gammaH2AX foci as an indication of lethal DNA lesions BMC Cancer 2010 10 4 10.1186/1471-2407-10-4 20051134 PMC2819996

[B35] Boeckman HJ Trego KS Turchi JJ Cisplatin sensitizes cancer cells to ionizing radiation via inhibition of nonhomologous end joining Mol Cancer Res 2005 3 277 285 10.1158/1541-7786.MCR-04-0032 15886299 PMC2432110

[B36] Dolling JA Boreham DR Brown DL Mitchel RE Raaphorst GP Modulation of radiation-induced strand break repair by cisplatin in mammalian cells Int J Radiat Biol 1998 74 61 69 10.1080/095530098141735 9687976

[B37] Pani E Stojic L El-Shemerly M Jiricny J Ferrari S Mismatch repair status and the response of human cells to cisplatin Cell Cycle 2007 6 1796 1802 10.4161/cc.6.14.4472 17622800

[B38] Takahashi M Koi M Balaguer F Boland CR Goel A MSH3 mediates sensitization of colorectal cancer cells to cisplatin, oxaliplatin, and a poly(ADP-ribose) polymerase inhibitor J Biol Chem 2011 286 12157 12165 10.1074/jbc.M110.198804 21285347 PMC3069420

[B39] Aebi S Kurdi-Haidar B Gordon R Cenni B Zheng H Fink D Christen RD Boland CR Koi M Fishel R Howell SB Loss of DNA mismatch repair in acquired resistance to cisplatin Cancer Res 1996 56 3087 3090 8674066

[B40] Drummond JT Anthoney A Brown R Modrich P Cisplatin and adriamycin resistance are associated with MutLalpha and mismatch repair deficiency in an ovarian tumor cell line J Biol Chem 1996 271 19645 19648 10.1074/jbc.271.33.19645 8702663

[B41] Duckett DR Drummond JT Murchie AI Reardon JT Sancar A Lilley DM Modrich P Human MutSalpha recognizes damaged DNA base pairs containing O6-methylguanine, O4-methylthymine, or the cisplatin-d(GpG) adduct Proc Natl Acad Sci USA 1996 93 6443 6447 10.1073/pnas.93.13.6443 8692834 PMC39042

[B42] Mello JA Acharya S Fishel R Essigmann JM The mismatch-repair protein hMSH2 binds selectively to DNA adducts of the anticancer drug cisplatin Chem Biol 1996 3 579 589 10.1016/S1074-5521(96)90149-0 8807890

[B43] Yamada M O'Regan E Brown R Karran P Selective recognition of a cisplatin-DNA adduct by human mismatch repair proteins Nucleic Acids Res 1997 25 491 496 10.1093/nar/25.3.491 9016586 PMC146450

[B44] Brown R Hirst GL Gallagher WM McIlwrath AJ Margison GP van der Zee AG Anthoney DA hMLH1 expression and cellular responses of ovarian tumour cells to treatment with cytotoxic anticancer agents Oncogene 1997 15 45 52 10.1038/sj.onc.1201167 9233776

[B45] Fink D Zheng H Nebel S Norris PS Aebi S Lin TP Nehme A Christen RD Haas M MacLeod CL Howell SB In vitro and in vivo resistance to cisplatin in cells that have lost DNA mismatch repair Cancer Res 1997 57 1841 1845 9157971

[B46] Papouli E Cejka P Jiricny J Dependence of the cytotoxicity of DNA-damaging agents on the mismatch repair status of human cells Cancer Res 2004 64 3391 3394 10.1158/0008-5472.CAN-04-0513 15150090

[B47] Watanabe Y Koi M Hemmi H Hoshai H Noda K A change in microsatellite instability caused by cisplatin-based chemotherapy of ovarian cancer Br J Cancer 2001 85 1064 1069 10.1054/bjoc.2001.2037 11592780 PMC2375095

[B48] Strathdee G MacKean MJ Illand M Brown R A role for methylation of the hMLH1 promoter in loss of hMLH1 expression and drug resistance in ovarian cancer Oncogene 1999 18 2335 2341 10.1038/sj.onc.1202540 10327053

[B49] Samimi G Fink D Varki NM Husain A Hoskins WJ Alberts DS Howell SB Analysis of MLH1 and MSH2 expression in ovarian cancer before and after platinum drug-based chemotherapy Clin Cancer Res 2000 6 1415 1421 10778972

[B50] Neher TM Bodenmiller D Fitch RW Jalal S Turchi JJ Novel Irreversible Small Molecule Inhibitors of Replication Protein A Display Single Agent Activity and Synergize with Cisplatin Mol Cancer Ther 2011 10.1158/1535-7163.MCT-11-0303 PMC3191262 21846830

[B51] Saleh-Gohari N Helleday T Conservative homologous recombination preferentially repairs DNA double-strand breaks in the S phase of the cell cycle in human cells Nucleic Acids Res 2004 32 3683 3688 10.1093/nar/gkh703 15252152 PMC484186

[B52] Zhao N Zhu F Yuan F Haick AK Fukushige S Gu L Her C The interplay between hMLH1 and hMRE11: role in MMR and the effect of hMLH1 mutations Biochem Biophys Res Commun 2008 370 338 343 10.1016/j.bbrc.2008.03.082 18373977 PMC2443822

[B53] Her C Wu X Griswold MD Zhou F Human MutS homologue MSH4 physically interacts with von Hippel-Lindau tumor suppressor-binding protein 1 Cancer Res 2003 63 865 872 12591739

[B54] Vo AT Zhu F Wu X Yuan F Gao Y Gu L Li GM Lee TH Her C hMRE11 deficiency leads to microsatellite instability and defective DNA mismatch repair EMBO Rep 2005 6 438 444 10.1038/sj.embor.7400392 15864295 PMC1299302

