# Supplementary material for: MutS homologue hMSH5: role in cisplatin-induced DNA damage response
Source: Mol Cancer. 2012 Mar 8;11:10. doi: 10.1186/1476-4598-11-10 (PMC3325843; doi:10.1186/1476-4598-11-10)
Supplement: Additional file 1 — Figure S1 Representative images of CDDP-induced hMSH5 foci formation at 1, 6, and 24 hrs post treatment. Cells were treated with 10 μM CDDP for 2 hrs and hMSH5 foci formation was analyzed at indicated time points after treatment. Figure S2. Representative images of cisplatin-induced γ-H2AX foci formation in 293T, 293T/f-hMSH5, 293T/f-hMSH5Y742F, and 293T hMSH5 RNAi cells (48 hrs post transfection with pmH1P-Bsd/hMSH5 sh-2). (A) Untreated cells were examined in parallel to establish the basal levels of γ-H2AX signal in these cell lines. (B) Cells were treated with 10 μM cisplatin for 2 hrs followed by γ-H2AX foci analysis at 24 hrs after cisplatin removal. Nuclei are counterstained with DAPI and merged images are provided. Figure S3. Representative images of CDDP-induced γ-H2AX, hRad51, and hMSH5 foci formation in 293T and A549 cells. (A) Analysis of CDDP-triggered γ-H2AX and hMSH5 foci formation. (B) Analysis of CDDP-triggered γ-H2AX and hRad51 foci formation. (C) Analysis of CDDP-triggered hRad51 and hMSH5 foci formation. Consistent with hMSH5 cytoplasmic and nuclear distribution patterns, CDDP-induced hMSH5 foci appear to be present in both cytoplasm and nucleus, whereas CDDP-induced γ-H2AX and hRad51 foci are predominately nuclear. Arrows indicate potential overlaps of two different signals. Figure S4. Effects of hMSH5 Tyr742-to-Phe mutation on its interaction with hMSH4 and c-Abl. (A) Co-IP analysis of the interaction between hMSH5 cp-1 Y742F and hMSH4. The results indicated that hMSH5 cp-1 Y742F could interact with hMSH4 as efficient as hMSH5 cp-1. (B) Co-IP analysis of the interaction between hMSH5Y742F and c-Abl. hMSH5Y742F interacted with c-Abl in the same manner as hMSH5 did. [file 1476-4598-11-10-S1.PDF]

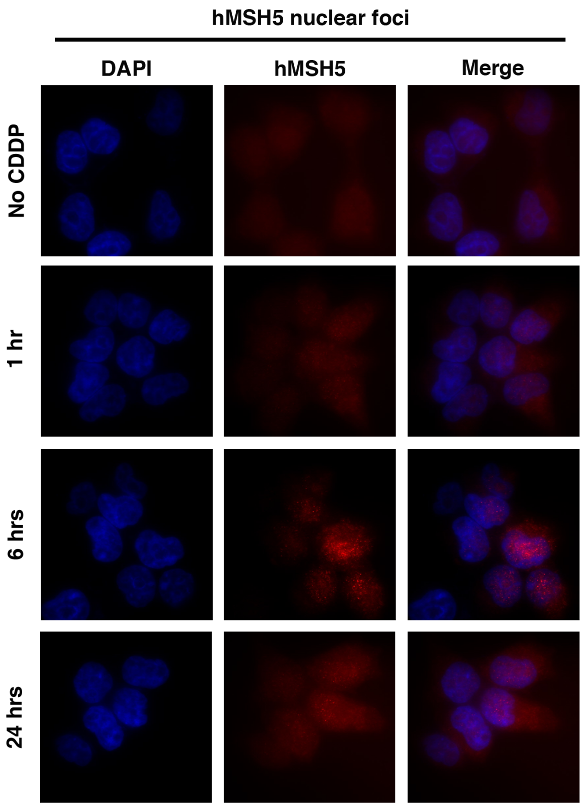

Fig. S1. Representative images of CDDP-induced hMSH5 foci formation at 1, 6, and 24 hrs post treatment. Cells were treated with 10 μM CDDP for 2 hrs and hMSH5 foci formation was analyzed at indicated time points after treatment.

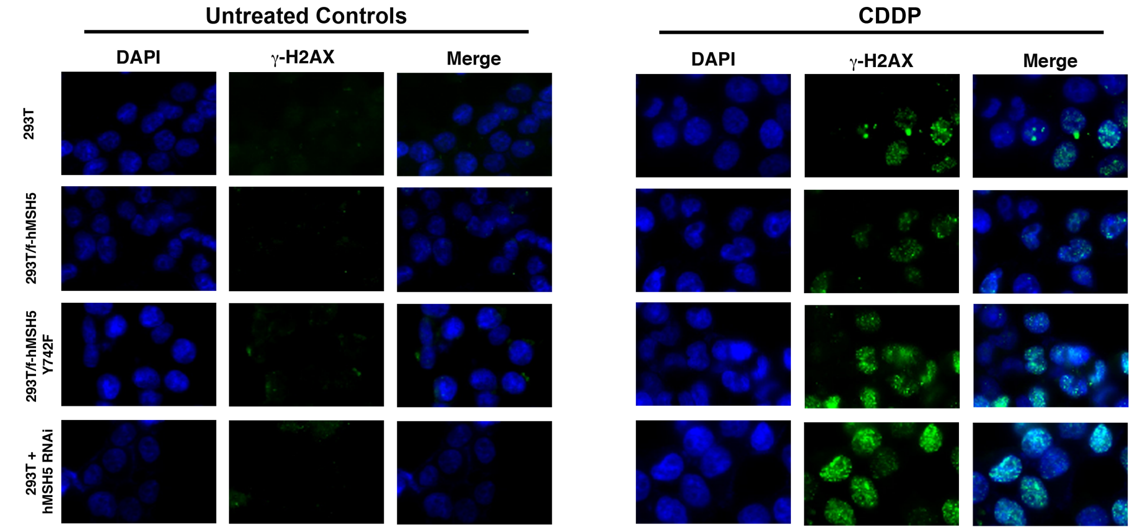

Fig. S2. Representative images of cisplatin-induced γ-H2AX foci formation in 293T, 293T/f-hMSH5, 293T/f-hMSH5^{Y742F}, as well as 293T hMSH5 RNAi cells (cells subjected to RNAi-mediated hMSH5 silencing; 48 hrs post transfection with pmH1P-Bsd/hMSH5 sh-2). (A) Untreated cells were examined in parallel to establish the basal levels of γ-H2AX signal in these cell lines. (B) Cells were treated with 10 μM cisplatin for 2 hrs followed by γ-H2AX foci analysis at 24 hrs after cisplatin removal. Nuclei are counterstained with DAPI and merged images are provided.

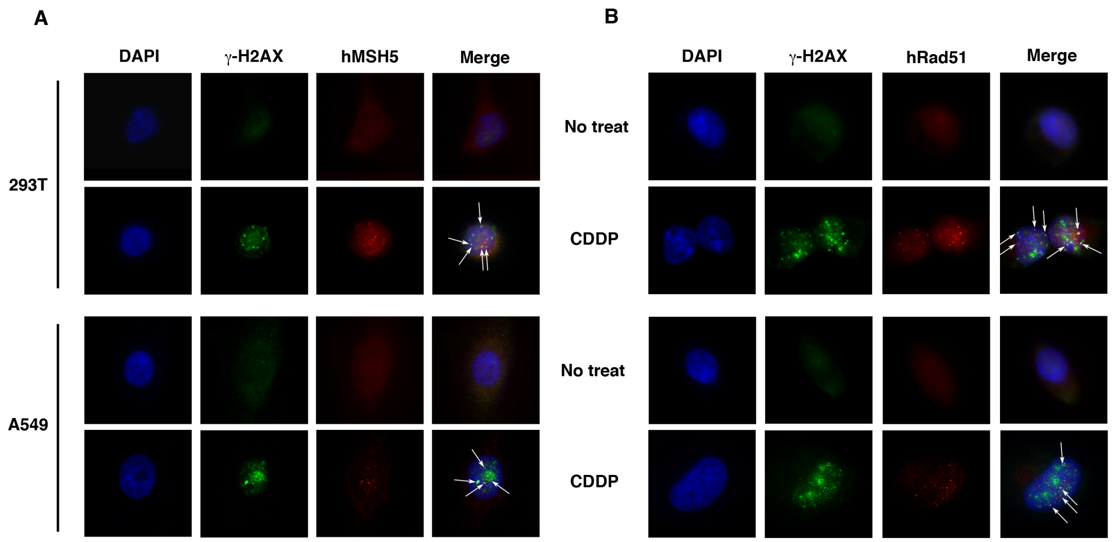

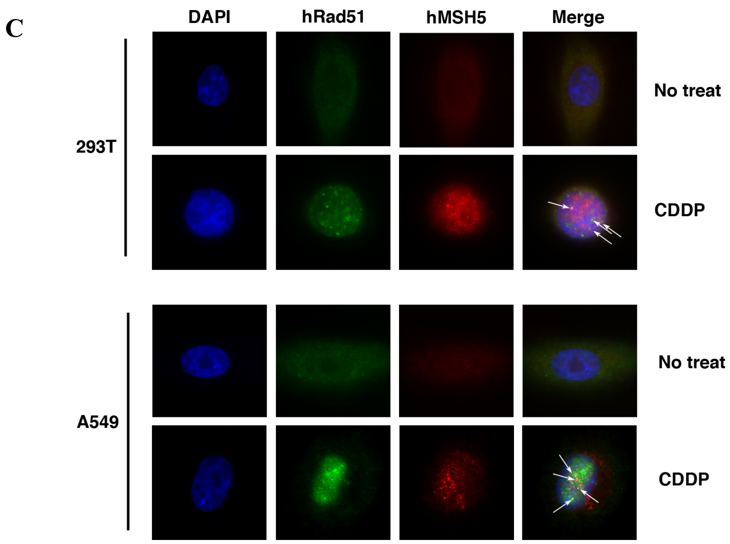

Fig. S3. Representative images of CDDP-induced γ-H2AX, hRad51, and hMSH5 foci formation in 293T and A549 cells. (A) Analysis of CDDP-triggered γ-H2AX and hMSH5 foci formation. (B) Analysis of CDDP-triggered γ-H2AX and hRad51 foci formation. (C) Analysis of CDDP-triggered hRad51 and hMSH5 foci formation. Consistent with hMSH5 cytoplasmic and nuclear distribution patterns, CDDP-induced hMSH5 foci appear to present in both cytoplasm and nucleus, whereas CDDP-induced γ-H2AX and hRad51 foci are predominately nuclear. Arrows indicate potential overlaps of two different signals.

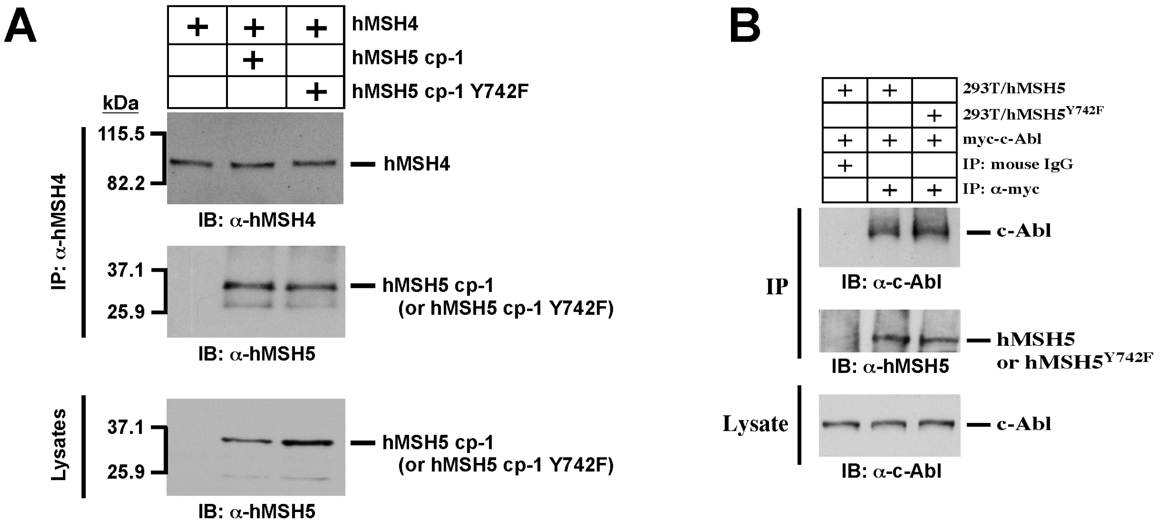

Fig. S4. Effects of hMSH5 Tyr^{742}-to-Phe mutation on its interaction with hMSH4 and c-Abl. (A) Co-IP analysis of the interaction between hMSH5 cp-1 Y742F and hMSH4. The results indicated that hMSH5 cp-1 Y742F could interact with hMSH4 as efficient as hMSH5 cp-1. (B) Co-IP analysis of the interaction between hMSH5^{Y742F} and c-Abl. hMSH5^{Y742F} interacted with c-Abl in a same manner as that of hMSH5.
